# O1-2 Identification of existing audit instruments for the assessment of urban and rural physical activity environments

**DOI:** 10.1093/eurpub/ckac094.002

**Published:** 2022-08-29

**Authors:** Bruno Domokos, Christina Müller, Lisa Paulsen, Izabela Bojkowska, Jens Bucksch, Birgit Wallmann-Sperlich

**Affiliations:** 1 Institute of Sport Science, University of Würzburg, Würzburg, Germany; 2 Department of Prevention and Health Promotion, Heidelberg University of Education, Heidelberg, Germany

**Keywords:** audit, assessment, community, neighbourhood, socio-ecological determinants

## Abstract

**Background:**

According to a socio-ecological perspective, the neighbourhood environment plays a critical role in promoting physical activity across all population groups. A comprehensive assessment of environmental characteristics is a prerequisite to the planning, implementation and evaluation of physical activity interventions. This work aims to identify existing audit tools for the assessment of physical activity related environmental features in order to evaluate a) their applicability in the European context and b) their relevance in the urban and rural context.

**Methods:**

Within the research project EUBeKo[1] we searched relevant websites (e. g. Active Living Research) and scientific databases (e. g. PubMed) in order to identify existing audit instruments. We then developed a categorization system for the integration of the topics and items retrieved from the identified tools. To reveal context-relevant strengths and weaknesses, we critically inspected the instruments with the support of the current scientific evidence and literature regarding eligibility to the European context and rural/urban applicability.

**Results:**

We identified 29 audit tools focusing on different topics and features influencing physical activity (e. g. infrastructure, barriers). Some also include aspects of the social environment (e. g. presence of people) or the subjective perception (e. g. walkability, aesthetics). We found that numerous tools contain aspects of the socio-ecological environment reflected on the item level that do not conform with European environmental characteristics (e. g. street signage, types of destinations). Furthermore, we identified an unequal distribution with few tools being developed explicitly for the rural context (n = 3).

**Conclusions:**

It is challenging to rate the applicability and relevance of audit tools. We were able to develop a comprehensive categorization system that can assist researchers and practitioners in the selection of a suitable set of items for an optimized assessment of European physical activity environments. In addition, our work points out the need for an instrument reflecting urban and specifically rural peculiarities of the European context.

1. The main purpose of the research project EUBeKo is to understand and systemize decision and implementation processes of policies and projects aiming to promote physical activity in the context of the built environment.

